# Moderate Rainfall and High Humidity During the Monsoon Season, Negligence in Using Malaria Protection Methods and High Proportion of Mild Symptomatic Patients Were the Driving Forces for Upsurge of Malaria Cases in 2018 Among Tea Tribe Populations in Endemic Dolonibasti Health Sub-center, Udalguri District, Assam State, North-East India

**DOI:** 10.3389/fmed.2022.913848

**Published:** 2022-06-30

**Authors:** Rahim Ali Ahmed, Hari Shankar, Syed Shah Areeb Hussain, Ananta Swargiary, Avdhesh Kumar, Mohammad Tarique, Pankaj Prabhakar, Harpal Singh Suri, Kuldeep Singh, Joy Kumar Chakma, Jyoti Singh, Afluza Begum

**Affiliations:** ^1^National Vector Borne Disease Control Programme, Guwahati, India; ^2^Parasite-Host Biology Group, ICMR – National Institute of Malaria Research, New Delhi, India; ^3^Indian Council of Medical Research, New Delhi, India; ^4^Department of Zoology, Bodoland University, Kokrajhar, India; ^5^National Vector Borne Disease Control Programme, Ministry of Health & FW, Government of India, New Delhi, India; ^6^Department of Child Health, University of Missouri, Columbia, MO, United States; ^7^Department of Pharmacology, Indira Gandhi Institute of Medical Sciences, Sheikhpura, India; ^8^Epidemiology & Environmental Biology Group, ICMR—National Institute of Malaria Research Field Station, Guwahati, India; ^9^Department of Zoology, Maitreyi College, University of Delhi, New Delhi, India; ^10^Department of Chemistry, Bhattadev University, Guwahati, India

**Keywords:** *Anopheles*, climate, community participation, malaria, outbreak

## Abstract

Malaria elimination is a global priority, which India has also adopted as a target. Despite the malaria control efforts like long-lasting insecticidal nets distribution, rounds of indoor residual spray, the introduction of bi-valent rapid diagnostic tests and artemisinin combination therapy, malaria remained consistent in Dolonibasti sub-center of Orang block primary health center (BPHC) under the district Udalguri, Assam state followed by abrupt rise in cases in 2018. Therefore, we aimed to investigate the factors driving the malaria transmission in the outbreak area of Dolonibasti sub-center. Malaria epidemiological data (2008–2018) of Udalguri district and Orang BPHC was collected. The annual (2011-2018) and monthly (2013–2018) malaria and meteorological data of Dolonibasti sub-center was collected. An entomological survey, Knowledge, Attitude and Practices study among malaria cases (*n* = 120) from Dolonibasti was conducted. In 2018, 26.1 % (2136/ 8188) of the population of Dolonibasti were found to be malaria positive, of which 55% were adults (*n* = 1176). Majority of cases were from tea tribe populations (90%), either asymptomatic or with fever only, 67.5 % (81/120) had experienced malaria infection during past years. The outbreak was characterized by a strong increase in cases in June 2018, high proportion of slide falciparum rate of 26.1% (other years average, 15.8%) and high proportion of *P. falciparum* of 81.2 % (other years average, 84.3%). *Anopheles minimus* s.l. was the major vector with 28.6% positivity and high larval density in paddy fields/ drainage area. Annual relative humidity was associated with rise in malaria cases, annual parasite incidence (r_s_ = 0.69, 90%CI; *p* = 0.06) and slide positivity rate (r_s_ = 0.83, 95%CI; *p* = 0.01). Older people were less educated (r_s_ = −0.66; *p* < 0.001), had lesser knowledge about malaria cause (r_s_ = −0.42; χ^2^=21.80; *p* < 0.001) and prevention (r_s_ = −0.18; *p* = 0.04). Malaria control practices were followed by those having knowledge about cause of malaria (r_s_ = 0.36; χ^2^ = 13.50; *p* < 0.001) and prevention (r_s_ = 0.40; χ^2^ = 17.71; *p* < 0.001). Altogether, 84.6% (44/52) of the respondents did not use protective measures. We described a sudden increase in malaria incidence in a rural, predominantly tea tribe population group with high illiteracy rate and ignorance on protective measures against malaria. More efforts that are concerted needed to educate the community about malaria control practices.

## Introduction

Malaria is a significant public health burden globally. Despite a considerable global effort to eradicate malaria in the last few years, the disease burden was 241 million in 2020, with 627000 deaths (1). The WHO African Region in 2020 contributes 95%, and the WHO South-East Asia Region accounted for about 2% of the global cases (1). India accounted for 83% of cases in the South-East Asia Region (1). The incidence of malaria declined worldwide globally in the endemic areas from 81 in 2000 to 56 cases per 1000 population in 2019, with a slight increase to 59 cases per 1000 in 2020 due to disruption of malaria management services during COVID-19 pandemic. The WHO South-East Asia Region witnessed a decrease in observed malaria incidence from 18 cases per 1000 population in 2000 to about three cases per 1000 in 2020 (~ 83% decrease) (1). It is not known if this decrease is due to decrease in disease incidence or disruption of non-Covid-19 health services, leading to missed diagnosis either due to common symptoms of malaria with Covid-19 or non-reporting of malaria cases due to overburdened Covid-19 cases (2). Recent study has also shown for dengue, that the Covid-19 interventions had a major impact in reduction of disease burden (3) and it is reasonable to assume similar phenomena for other vector borne diseases like malaria. Notably, this is a global phenomenon and disease surveillance post-Covid-19 needs to be developed and targeted to address such issues (4).

Malaria elimination and eradication are global priorities (5). The control strategies are at the fore-front globally, including but not limited to antimalarial drug development, diagnostics, vaccine development, and vector management. India has also adopted malaria elimination as a target by 2030 (6, 7); therefore, launched the National Framework of Malaria Elimination in 2016, followed by the Malaria Elimination Research Alliance (MERA)-India in 2019 to focus on operational and implementation research (8, 9). In addition, malaria data integration platforms using real-time epidemiological, entomological, and commodity surveillance data to formulate malaria control policies are also likely to be adopted soon (8). However, despite the efforts, malaria is still one of the most significant public health concerns in India (10, 11). In India, malaria transmission is predominantly due to either *P. falciparum* or *P. vivax* (12). However, the prevalence of these species varies from one region to another. There has been a steady increase in *P. falciparum* cases in the last four decades. By December 2018, *P. falciparum* was the major malarial parasite responsible for 48.1% of the malaria cases in India, and rest of the cases either was due to *P. vivax* or mixed infections of *P. falciparum* and *P. vivax* (13). North-East India, Odisha, Chhattisgarh and Jharkhand contribute most of the *P. falciparum* cases (78.6%) in the country, of which NE India alone contributes 12.4% of *P. falciparum* and 1.2% of *P. vivax* cases in India (13). The common clinical symptoms observed in the patients with *P. falciparum* and *P. vivax* is fever, chills, headache, sweating, myalgia/ arthralgia.

Malaria epidemiology in the North-Eastern states of India viz. Arunachal Pradesh, Assam, Manipur, Meghalaya, Mizoram, Nagaland, Sikkim and Tripura showed a steady decline in malaria cases of Assam from 89,939 (*P. falciparum*−58,124) in 2008 to 3816 (*P. falciparum*−2859) in 2018 (95.8% decline). Based on the topography and socio-demographic conditions, malaria in NE India was classified into four different categories viz. tribal malaria, forest malaria, border malaria, and organized sector malaria (14). In 2018, the observed malaria cases in Assam were relatively low as compared to other North-Eastern states such as Tripura (13,079) and Meghalaya (6394). The *P. vivax* cases also declined continuously in Assam state from 25,815 in 2008 to 957 in 2018 (96.3% decline). As of 2018, *P. falciparum* and *P. vivax* contribute 74.9% and 25.1% of the total cases in Assam (13). There are 27 districts in Assam, and district Udalguri reported 2371 malaria cases in 2018, contributing 62.1% (2371/3816) of state total malaria cases. Udalguri district of Assam state is located in the foothills of Arunachal Pradesh, neighboring the country of Bhutan. It is mainly covered by forest and tea gardens and is dominated by the tribal population (Bodo and Tea tribes). *P. falciparum* is the major malaria parasite in the Udalguri district and contributes to more than 80% of malaria cases. A surge in malaria cases was reported at Dolonibasti health sub-center under Orang Block Primary Health Center (BPHC) of Udalguri district in 2018. Such an abrupt rise may be attributed to a variety of factors like an increase in vector breeding sites, meteorological factors, migration of infected population into a vector-rich area having the susceptible population, arrival of new efficient vectors, inadequate vector control measures, resistance of mosquitoes to currently used insecticides and resistance of parasites to the antimalarial drugs (15).

Climate is a crucial driver of malaria transmission, affecting the survival and proliferation of mosquitoes malaria vectors. The climate of Assam is “Tropical Monsoon,” characterized by moderate temperatures, high humidity, and heavy rainfalls—conditions ideal for vector breeding (13). However, excess rain in the region can also lead to the overflowing of the Brahmaputra and the Barak rivers, flooding the nearby areas and causing significant damage to life and property (13). Prolonged periods of rainfall in the state support perennial transmission of malaria with a seasonal peak during April-September, corresponding to the monsoon period. The highly suitable climate makes Assam hyper-endemic for malaria, despite numerous intervention strategies that have been undertaken since the establishment of the National Malaria Control Programme in 1953 (15). Some of the critical intervention strategies adopted by the Programme are active surveillance of malaria cases, periodic administration of Indoor Residual Spray, distribution of Long-lasting insecticidal nets/ Insecticide-treated bed nets (LLINs/ITNs), distribution of anti-malarial drugs and improving the knowledge and practices of the community through Information Education Communication (IEC) and Behavioral Change Communication (BCC) activities (16, 17).

While these interventions helped reduce malaria in several parts of Assam over the past decade, they have largely failed to produce any significant effect in some regions, such as the Dolonobasti sub-center under Orang BPHC in Udalguri district (population of 8,188, area of 20.3 Sq. Km) that witnessed a sharp rise of malaria cases in 2018. This study aimed to investigate the possible factors involved in the emergence of these malaria cases in 2018, as well as the persistence of malaria in the region despite the intervention efforts. This would enable the local authorities to initiate the malaria contraction plan in the affected area and increase their preparedness and response time to tackle such situations.

## Materials and Methods

### Study Area

**Figure 1:**
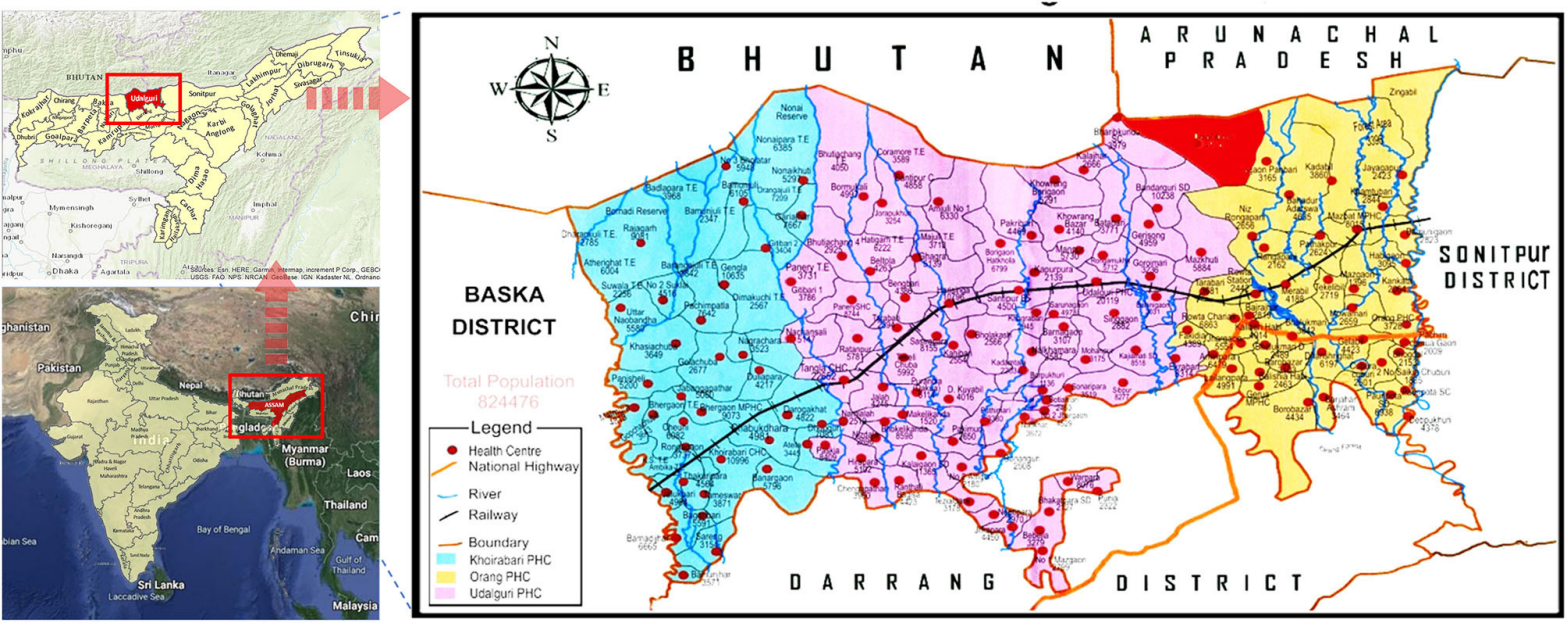
Map of study area (Red color depicts the area covered under the Dolonibasti sub-center).

The Dolonibasti sub-center is covered under the Orang BPHC of the district Udalguri, Assam state and is located at Latitude: 26° 51' 16.50“ N, Longitude: 92° 10' 48.20” E and an altitude of about 345 meters above sea level (Figure 1). There are seven villages namely Dolonibasti, Sikaridanga, Onthaibari, Bogoribari, Thakurpara, Lalpani, NK Monai and one Tea estate (Dhansiri Tea Estate including Pahartoli) under the Dolonibasti sub-center. The area with upsurge in malaria cases was defined as the whole Dolonibasti sub-center. The Dolonibasti sub-center had a population of 8188 as per the National Vector Borne Disease Control Programme (NVBDCP) annual census 2018 of the district and covers an area of 20.3 Sq. Km. This study is a retrospective analysis of the data collected by the NVBDCP for the period of 2008-2018.

### Epidemiological Data

A district is categorized by the Block Primary Health Centers (BPHCs). Each BPHC covers a few sub-centers. Each sub-center covers 6–7 villages based on the average population coverage of nearly 5500. Following the abrupt rise of malaria cases in June 2018, epidemiological data of each of the seven villages under the Dolonibasti sub-center was obtained from Orang BPHC, and the Udalguri district unit of NVBDCP. There are three BPHCs under the district Udalguri, namely Udalguri, Orang and Khoirabari. Malaria epidemiological data (2008-2018) was obtained for the entire district and its three BPHCs. Annual malaria data for the Dolonibasti sub-center was obtained for the period of 2011-2018. To conduct a detailed investigation about the factors responsible for the surge in malaria cases in 2018, month-wise, malaria data of the Dolonibasti sub-center was retrieved from 2013 through 2018. Due to a sudden rise in malaria cases in 2018, the test and treat strategy was scaled-up in the entire district, and for each village, one healthcare worker was assigned per approximately 2,000 population. However, before 2019, one healthcare worker was appointed to over 8,000 population. Age and gender information (*n* = 2,130) was also retrieved from 2018 line list of positive malaria cases (*n* = 2,136). The geographic locations of the villages covered under the Dolonibasti sub-center revealed that the region was densely forested, at the foothills and surrounded by hill streams, rice fields and tea gardens/plantages.

### Entomological Survey

#### Adult Survey

The entomological survey was conducted in randomly selected households of affected villages following the standard method (18). The adult and larval forms of mosquitoes were collected only from those villages where malaria positivity was high compared to other villages of Dolonibasti sub-center. The four villages surveyed for entomological investigations were Dolonibasti, Sikaridanga, Lalpani, NK Monai and Dhansiri Tea Estate. Indoor resting adult mosquitoes were collected in the morning between 4 to 6 AM and evening between 6 to 8 PM using a torch and suction tube (18). Each house was surveyed for 15 min (18). The mosquitoes were identified under the microscope using standard identification keys (6, 7) and Per Man Hour Density (PMHD) of each mosquito species was calculated using formula PMHD = Total no. of mosquitoes collected/No. of person X Time spent).

#### Larval Survey

During the survey, larvae were collected from different water bodies like drainage sources in residential areas, agricultural and tea estate canals, paddy fields and other containers present in that area using the dipping method. Collected larvae from a different source were kept separately in plastic containers. The genus of larvae was identified in the field using standard identification key (19, 20).

#### Mosquitoes Dissection

Mosquito dissection was conducted with standard methods (18). Adult female mosquitoes were anesthetized to assess the sporozoite and oocyst infections in the salivary glands and midgut of *An. minimus* s.l and *An. dirus* s.l. A drop of phosphate buffer saline was placed on a clean microscope slide, and salivary glands were detached from the thorax by holding the mosquito with forceps. Similarly, unfed female mosquitoes' gut were separated. The dissected salivary glands and gut were covered with a coverslip, gently pressed, and observed under a microscope at 400x resolution.

### Meteorological Data

To analyze the role of meteorological factors on malaria, annual (2011–2018) and monthly (January, 2013–December, 2018) data of temperature, rainfall and relative humidity was extracted for the point location of the Dolonibasti sub-center. Monthly and annual temperature data at 0.1° x 0.1° resolution was obtained from the FLDAS Noah Land Surface Model (21), precipitation data at 0.1° x 0.1° resolution was accessed from the Climate Hazards Group InfraRed Precipitation and Station data (CHIRPS) dataset (22) and relative humidity data at 0.25° x 0.25° resolution was obtained from the Climatic Research Unit gridded Time Series datasets (23). Since, the exact coordinates of the affected households were not available; therefore, the computation was done by taking averages.

### Knowledge, Attitude and Practices (KAP)

The investigation was expanded by conducting a cross-sectional study at the end of 2018 using a structured questionnaire about positive malaria cases' knowledge, attitude, and practices related to malaria control practices. Our previous active case surveillance study in 2017 reported nearly 8.1% (681/8411) proportion of asymptomatic *Plasmodium* infection and 5.1% (145/2818) of the cases with low-density *Plasmodium* infection in the district Udalguri, Assam state and East Garo Hills, Meghalaya state of NE India (24). Since, asymptomatic and low-density infections are the main reservoir of *Plasmodium* infection and contribute malaria persistence in the region; therefore, sample size for KAP study was calculated by considering the proportion of asymptomatic cases/low-density infection cases. The sample size was calculated using a population size of 2,136 with the accuracy of 5.2% and a sampling error rate of 26.7 at 95% Confidence Interval (CI). This resulted in a sample size of 120 that would suffice to achieve the required level of accuracy. A total of 120 persons were selected through random sampling from the line list of positive malaria cases (*n* = 2,136) detected in 2018 in the Dolonibasti sub-center. The questionnaire included recall of symptoms of their own cases, the perception of people about mosquitoes, their breeding places, various diseases spread by them, control measures and personal protection measures in the community and source of treatment.

### Statistical Analysis

The impact of annual climatic trends (2011–2018) and monthly seasonality (2013–2018) on malaria persistence in Dolonibasti sub-center was assessed using scatter plots and Spearman's rank correlation. The responses obtained from the KAP survey were converted to frequency distributions and cross-tabulated in Microsoft Excel. Spearman's correlation and Chi-square test were applied to identify the relationship between the various KAP variables and Benjamini and Hochberg correction was applied in order to account for multiple testing. The KAP variables showing significant association were plotted in Mosaic plots (25) to analyze the magnitude and type of association. All the statistical calculations were performed in statistical software R 3.4.3 for Windows (R Project for Statistical Computing) and Origin Pro-8 software (OriginLab Corporation, Northampton, USA). The critical value for statistical significance was considered at alpha 0.05.

## Results

**Figure 2:**
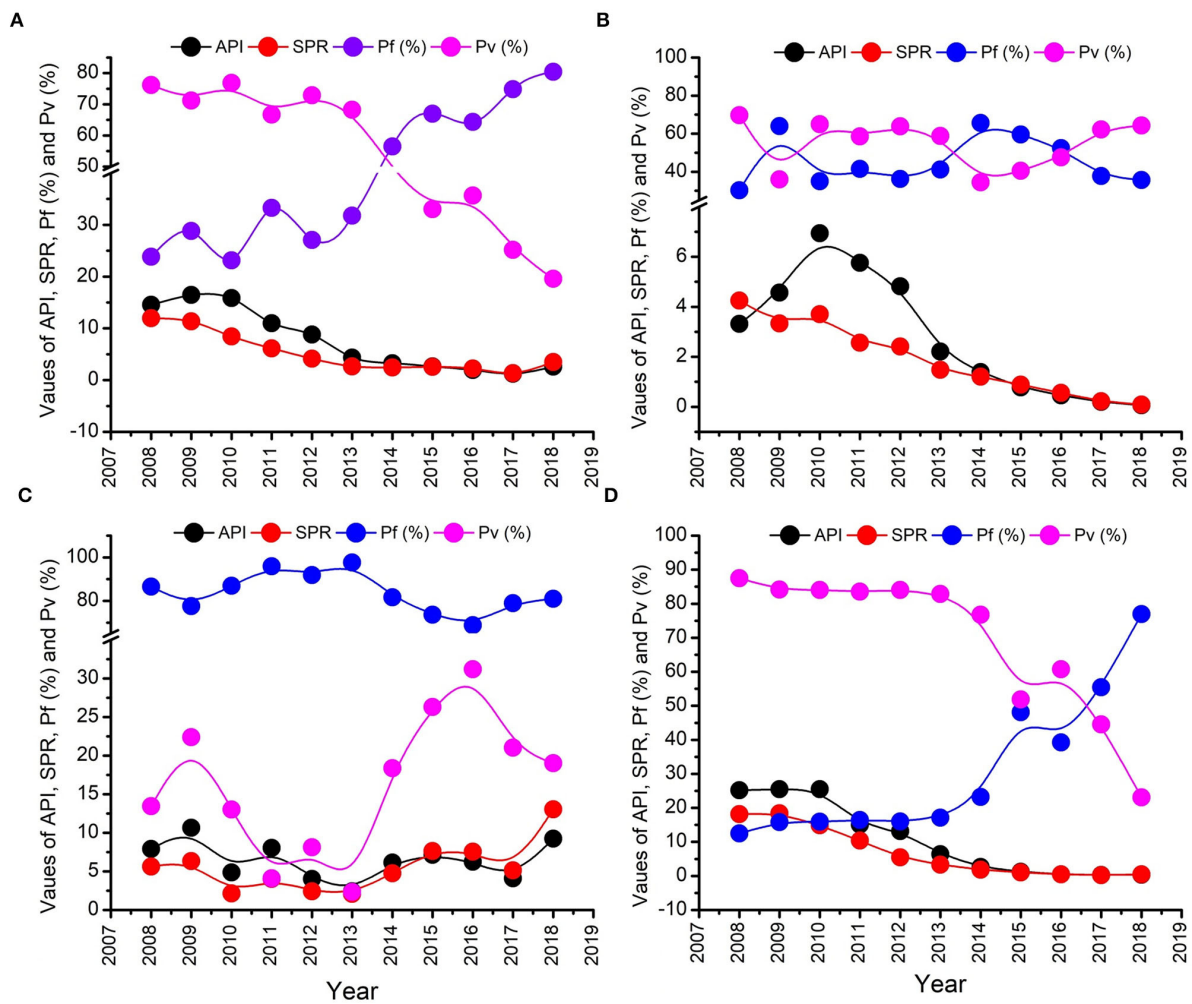
Malaria epidemiological trend from 2011 to 2018. **(A)** Entire Udalguri district, **(B)** Khoirabari BPHC, **(C)** Orang BPHC, and **(D)** Udalguri BPHC.

Annual parasite incidence is the number of confirmed malaria cases registered in a specific year, expressed per 1,000 individuals under surveillance. The malaria epidemiological data of the Udalguri district showed a high annual parasitic incidence of 14.5 in 2008, which was declined to 2.6 in 2018, followed by a further decline up to 0.01 in 2021 (Supplementary Table 1). District Udalguri (Population 9,09,442, NVBDCP Census 2018) encompasses 153 health sub-centers under 3 Block PHCs viz. Udalguri, Khoirabari and Orang. Between 2008 and 2018, though the annual parasite incidence and slide positivity rate in district Udalguri gradually decreased, the *P. falciparum*% increased from 23.8 in 2008 to 80.4 in 2018 (Supplementary Table 1, Figure 2A). While the malaria caseload in BPHCs Udalguri and Khoirabari gradually decreased between 2008 and 2018 (Figures 2B,D), the Orang BPHC maintained high malaria endemicity (Figure 2C) with a rising trend. In 2018, district Udalguri witnessed a spurt and recorded 2,371 malaria cases, 92.8% of which (2,201 cases) were contributed by the Orang BPHC alone. The rise in malaria prevalence in 2018 was 56% over 2017, 33.7% in 2016 and 23.5% over 2015 (Supplementary Table 2). After 2019, there is a continued decline in malaria cases in the district and Dolonibasti sub-center.

### Epidemiological Observations in Dolonibasti Sub-center

Upon observing the epidemiological data, it was found that a total of 2,136 malaria cases, about 97% of Orang BPHC and 91% of the district's total cases, were reported from the Dolonibasti sub-center. Dolonibasti sub-center is located at the foothills of Arunachal Pradesh, India, and comes under Orang BPHC of Udalguri District. The population of the Dolonibasti sub-center was 8188 as per NVBDCP Annual Census 2018, and a majority of the population is Tea tribes (74%), followed by Bodo Tribes (23%) and others (3%). A high proportion of population was infected, overall about 26.1% (2136/8188), assuming one infection per person. The malaria incidence in the Dolonibasti sub-center during 2011–18 is given in Table 1. In the Dolonibasti sub-center, the annual blood examination rate in 2011 was 85.8%, ranging between 47 to 81% during 2012–18. Malaria cases decreased during 2013 and again increased in 2014 but remained stable during 2015–16 (Table 1). The slide positivity rate fell from 18.8 in 2011 to 12.3 in 2013. However, from 2013 onwards, the slide positivity rate increased and reached 32.2 in 2018. The infection in the area was due to *P. falciparum* and *P. vivax*. Two cases of mixed infection (both *P. falciparum* and *P. vivax*) were also reported in 2018 from the Dolonibasti sub-center, the outbreak region. Similarly, the Slide Falciparum Rate also increased, and in 2018, it was 26.1, which was the highest of the study period. It was also observed that the *P. falciparum* infection was more (70–98%) in comparison to *P. vivax* (2-30%) at any period of time (Table 1). In the outbreak year 2018, Dolonibasti sub-center encountered exceptionally high number of *P. falciparum* cases (1734, other years with an average case of 813) and proportion of 81.2% (other years with an average of 84.3%). Furthermore, during 2018, the slide positivity rate (reflecting prevalence) was particularly high (32.2%) in the hotspot area of Dolonibasti sub-center with annual blood examination rate of 81.0%, and Orang Block PHC under which outbreak sub-center falls had slide positivity rate of 13.0%, while its annual blood examination rate was only 7.1%.

**Table 1:** Malaria epidemiology in the Dolonibasti sub-center.

**Year**	**Population**	**BSC/E**	**Total positive**	**Pf**	**Pv**	**ABER**	**API**	**SPR**	**SFR**	**Pf%**	**Pv%**	**Death**
2011	6,837	5865	1,100	1047	53	85.78	160.89	18.76	17.85	95.18	4.82	0
2012	6,878	4179	753	695	58	60.76	109.48	18.02	16.63	92.30	7.70	0
2013	7,013	3309	407	399	8	47.18	58.04	12.30	12.06	98.03	1.97	0
2014	7,224	7218	1,067	896	171	99.92	147.70	14.78	12.41	83.97	16.03	0
2015	7,994	5911	1,326	953	373	73.94	165.87	22.43	16.12	71.87	28.13	0
2016	8,073	5635	1,389	955	434	69.80	172.05	24.65	16.95	68.75	31.25	0
2017	8,150	4044	934	746	188	49.62	114.60	23.10	18.45	79.87	20.13	0
2018	8,188	6633	2,136	1734	402	81.01	260.87	32.20	26.14	81.18	18.82	0
2019	8,271	6218	314	230	84	75.18	37.96	5.05	3.70	73.25	26.75	0
2020	8,345	5146	73	43	30	61.67	8.75	1.42	0.84	58.90	41.10	0
2021	8,496	5023	8	3	5	59.12	0.94	0.16	0.06	37.50	62.50	0

*Pf, Plasmodium falciparum; Pv, Plasmodium vivax; ABER, Annual Blood Examination Rate; API, Annual Parasitic Incidence; SPR, Slide Positivity Rate; SFR, Slide Falciparum Rate*.

**Figure 3:**
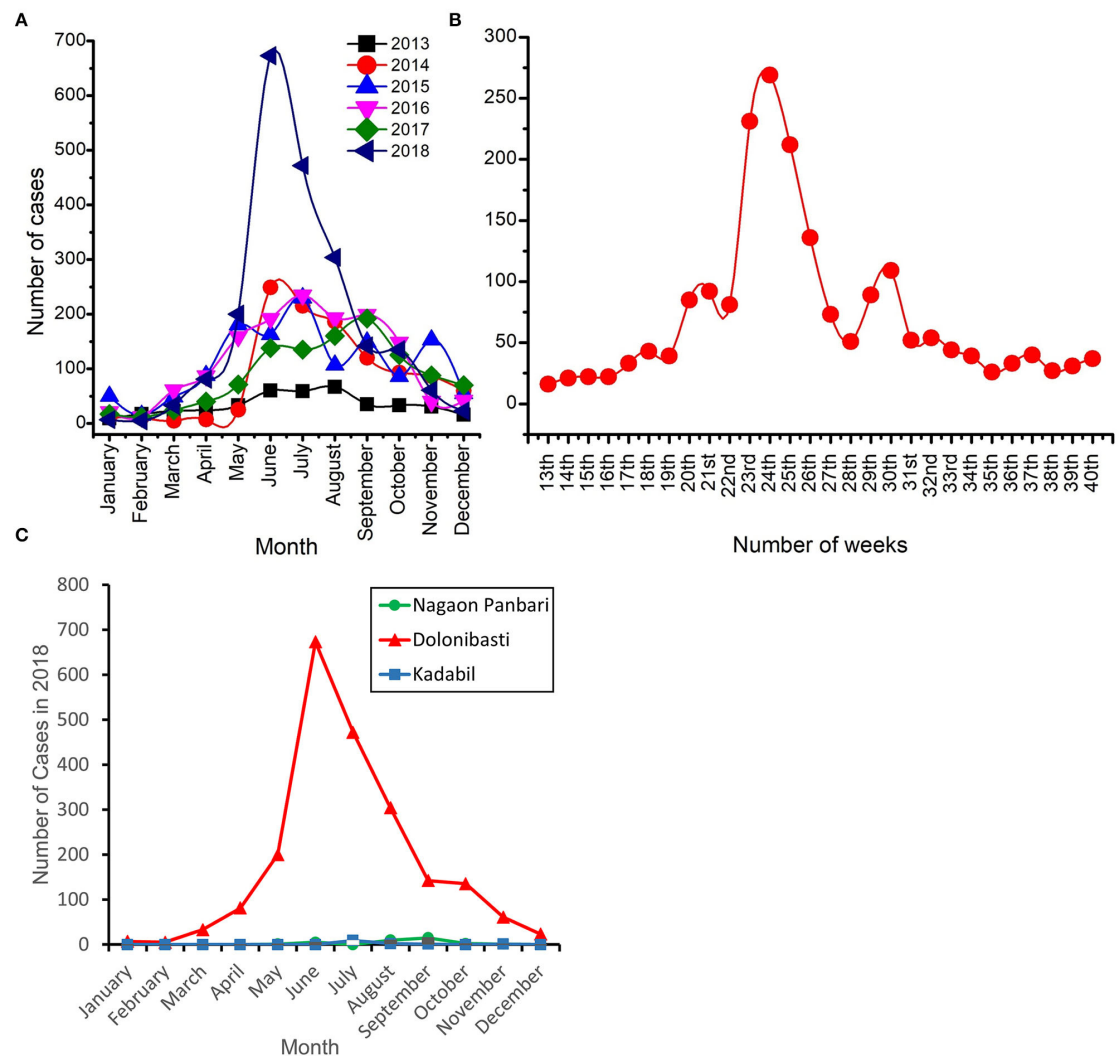
Number of malaria cases reported in the Dolonibasti sub-center and its adjoining two sub-centers of Orang BPHC. **(A)** Month wise incidence of malaria cases during 2013–18. **(B)** Weekly incidence of malaria cases during 2018 and **(C)** Month wise incidence of malaria cases during 2018 in the Dolonibasti sub-center and it's adjoining two sub-centers namely Nagao Panbari and Kadabil.

Monthly incidence of malaria cases in the Dolonibasti sub-center showed that the cases started to rise in April 2018 and reached its peak in June 2018 (Figure 3A). A total of 40 malaria cases were reported in April 2017, while it was doubled to 81 in 2018. Furthermore, by the end of June 2018, the malaria cases rose to 673, 79.5% more than in 2017 (*n* = 138), 71.5% more than in 2016 (*n* = 192) and 75.8% more than in 2015 (*n* = 163), Figure 3A. Weekly fluctuations in the number of malaria cases during 2018 are shown in Figure 3B. The peak of the cases was observed between 23rd−25th weeks corresponding to the months of June-July of the year 2018. Monthly incidence of malaria cases in 2018 reported from the Dolonibasti sub-center and it's adjoining two sub-centers, namely Nagaon Panbari and Kadabil is shown in Figure 3C. The adjoining two sub-centers reported negligible malaria cases in comparison to the cases reported from the Dolonibasti sub-center.

### Age and Sex-Wise Distribution of Malaria Cases in Dolonibasti Sub-center

It was observed that the majority of malaria cases (55.2%) were above 14 years of age (1176/2130), followed by 28.6% from 5–14 years of age (609/2130) and children (16.2%) of age-group 0–4 years (345/2130). Both the genders shared nearly an equal proportion of cases.

### Entomological Observations in Dolonibasti Sub-center

A total of 1005 adult mosquitoes belong to five genera, and 25 species were caught from the Dolonibasti sub-center. Out of 25 species, 11 (44%) belonged to the *Anopheles* genus. In terms of numbers of mosquitoes collected, *Culex* was highest with 528 (52.5%), followed by *Anopheles* 230 numbers (22.9%) and 247 species (24.6%) of other mosquito species from the genus *Mansonia, Armigeres* and *Aedes*. *An. Minimus* s.l.*, An. dirus* s.l., *An. culicifacies* s.l.*, An. fluviatilis* s.l., and *An. barbirostris* s.l. were abundant in the Dolonibasti sub-center. The PMHD of the primary vector *An. minimus* s.l. was 1.70, which was higher than *An. barbirostris* s.l. (1.25), *An. dirus* s.l. (0.43), *An. culicifacies* s.l. (0.33) and *An. Fluviatilis* s.l. (0.18) (Supplementary Table 3). The study observed that the hanging clothes were the preferred resting sites of *An. Minimus* s.l. in the human dwelling. Since, *An. minimus* s.l. and *An. dirus* s.l. are the primary vectors in the region (12); therefore, parasite positivity was checked only in these two vectors. A total of 68 *An. minimus* s.l. and 17 *An. dirus* s.l. mosquitoes were collected from the Dolonibasti sub-center. There was no positivity found in *An. dirus* s.l. while dissecting all 17 mosquitoes. Out of 68 *An. minimus* s.l., 29 were unfed and 39 were fully fed. Of 29 unfed mosquitoes, 28 were dissected for gland and gut positivity, and 28 out of 39 fully fed mosquitioes were dissected for gland positivity. The proportion of unfed and fully fed condition of major vector species (*An. Minimus* s.l.) was recorded as 42.8 (29/68) and 57.2 (39/68), respectively. The parasite positivity in the gland (16/56) and gut (8/28) of *An. minimus* s.l. was 28.6%.

Mosquito larvae collected from five different collection sites are shown in Table 2. Out of five different sites, the highest density of collected larvae was obtained from the paddy field, followed by the drainage system of the residential area. The larvae of the *Anopheles* mosquitos were prevalent in all the collection sites except agricultural canals.

**Table 2:** Larval density of mosquito species in the Dolonibasti sub-center.

**Breeding sites**	**Larva per dip**	**Species**	**Larval Density**
Drain in residential colony	11 ± 3.5	*Anopheles, Culex, Mansonia species*	Medium
Agricultural canal	9 ± 3.5	*Armigeres, Culex, Species*	Medium
Tea estate canal	10 ± 3.5	*Anopheles, Culex, Mansonia species*	Medium
Paddy field	16 ± 3.5	*Anopheles, Culex, Mansonia species*	Medium
Other containers	5 ± 3.5	*Aedes, Anopheles, Culex, Mansonia species*	Medium

*Density–Low, 1–4, Medium, 5–60 and High = >60*.

### Role of Meteorology in Malaria Persistence

**Figure 4:**
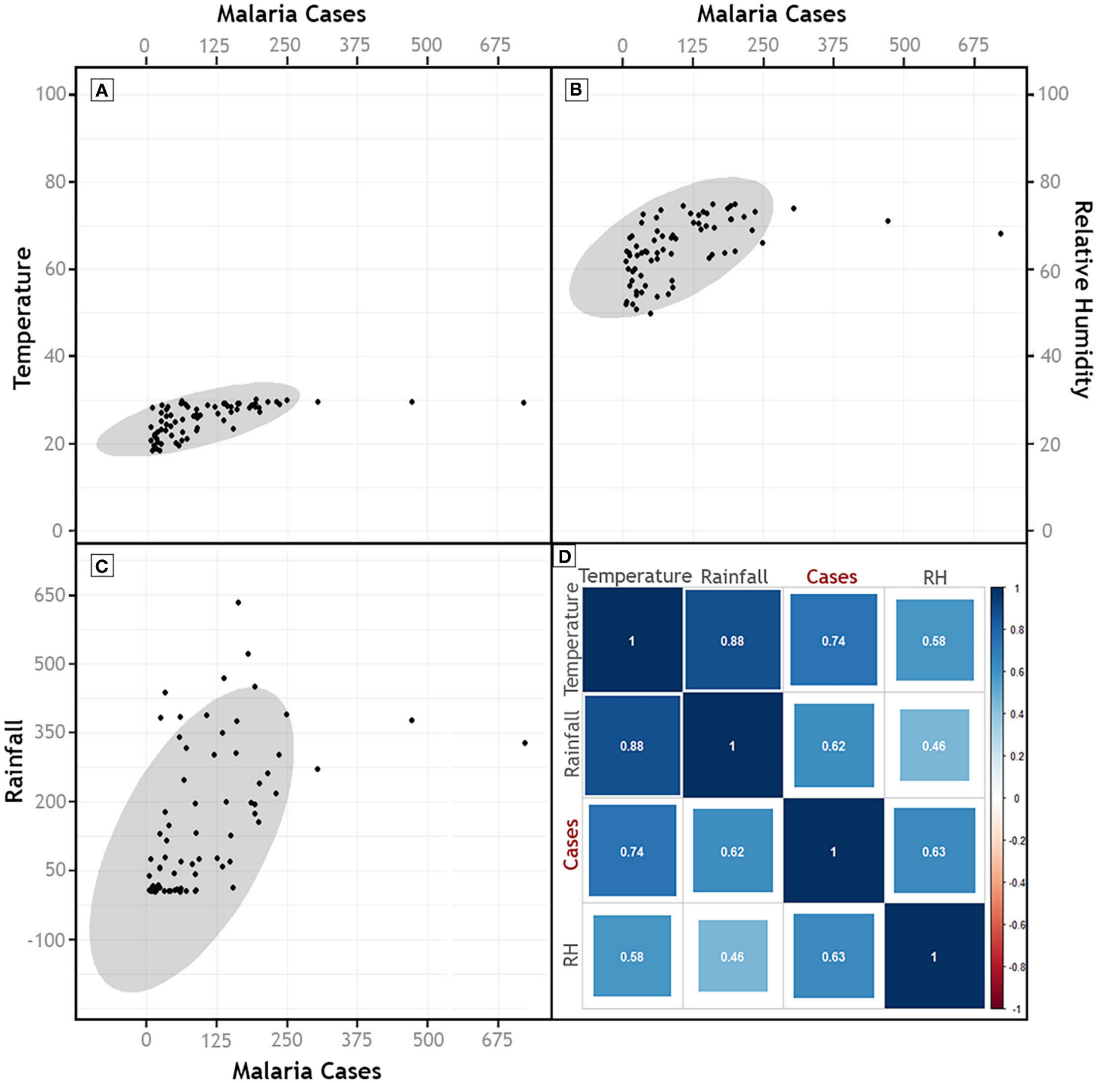
Scatter plots of Malaria Cases and **(A)** Temperature, **(B)** Relative Humidity, and **(C)** Rainfall. **(D)** Represents the results of Spearman's Correlation of malaria cases with monthly climatic parameters (2013–2018). Color of the correlation plot **(D)** indicates the magnitude of the association, whereas size of the box indicates statistical significance, with big boxes indicating high significance values.

Climatic trends and seasonality play a crucial role in determining the survival and abundance of arthropod vectors, as well as the incubation period of malaria parasites in the arthropod vectors. Annual variation of climatic factors (2011-2018), namely, temperature, rainfall and RH, did not show any discernible correlation with the malaria prevalence but a strong positive correlation was observed between relative humidity and slide positivity rate (r_s_= 0.83; *p* = 0.01) in Dolonibasti sub-center (Supplementary Tables 4, 5). The association studies revealed that peak malaria years in the Dolonibasti sub-center (outbreak region) occur during the monsoon season between the months of April to August, especially during the years when relative humidity is high, but rainfall remains at a relatively modest level. However, the monthly variation (2013-2018) of these climatic factors recorded strong (*p* < 0.001) positive correlation (Temperature- r_s_ = 0.74; Rainfall- r_s_ = 0.62; RH- r_s_ = 0.63) with the malaria prevalence (Supplementary Table 6, Figure 4).

### Knowledge, Attitude and Practices in Dolonibasti Sub-center

Among respondents (*n* = 120), 38.3% were female (*n* = 46) and rest were male. The median age of respondents was 33 years, with an interquartile range between 23- and 45 years. Nine respondents were children between 9- and 14 years and rests were above 15 years of age. Table 3 describes the demography of respondents and their knowledge, attitude and practices toward malaria. It was observed that 61.7% were illiterate but the average literacy of district Udalguri as per Census 2011 data is 65.4%, and about 15.8% were educated up to the upper primary level. Most of the respondents were daily-wage labors and worked in tea gardens. Their socioeconomic condition was low, with a daily income of INR 167/person. Most of the houses in the outbreak region were kutcha (made of clay, bamboo, flax, grass, crop residues, mulch and unburnt bricks) and semi-kutcha houses (roofs are generally made of hay, while the exterior walls are built with concrete), and only those living inside the tea garden had pucca houses (solid construction that is made up of bricks, iron, metal and other robust materials) allotted by the company to the tea garden workers.

**Table 3:** Knowledge, Attitude and Practices related to malaria among respondents (*n* = 120) in the Dolonibasti sub-center, 2018.

**Characteristics (*n* = 120)**	**No**.	**Percentage**
**Age-group of the respondent**		
9–14 year	9	7.50
15–25 year	38	31.67
26–50 year	56	46.67
51 years and above	17	14.17
**Educational status**		
Illiterate	74	61.67
Up to Primary	16	13.33
Up to Upper Primary	19	15.83
Up to High School	11	9.17
**Symptoms of malaria**		
No symptom	29	24.17
Fever only	46	38.33
Fever with chill	13	10.83
Headache	18	15.00
Body ache	7	5.83
Vomiting	6	5.00
Others	1	0.83
Don't know	0	0.00
**Treatment done**		
Government Agency	95	79.17
Private Agency	11	9.17
Others (Pharmacy/Quack)	14	11.67
**Hospitalization**		
Yes	2	1.67
No	118	98.33
**History of malaria**		
*Malaria episodes in previous year (2017)*
Yes	53	44.17
No	67	55.83
*Malaria episodes in last 2–3 years (2015-2017)*
Yes	81	67.50
No	39	32.50
**Cause of malaria**		
Correct	94	78.33
Wrong	26	21.67
**Knowledge on preventive measures for malaria**		
Correct	87	72.50
Wrong	33	27.50
**Anti-mosquito measures adopted**		
Yes	68	56.67
No	52	43.33
**Reason for not using anti-mosquito measures**		
Economic conditions	8	15.77
Do not know/answer	12	23.08
Ignorance	23	44.23
Do not care	9	17.31

Out of 120 cases, 72.5% were *P. falciparum* infections, and the rest were *P. vivax* infections. Twenty-nine (24.2%) respondents had asymptomatic malaria, of which 20 (69%) were *P. falciparum*, and 9 (31%) were *P. vivax* infections, whereas 46 (38.3%) had fever symptom only. Only 6 (5%) respondents experienced vomiting, and two respondents were admitted to the hospital due to severe malaria. The Government health delivery system diagnosed 80% of malaria cases through active surveillance (by Health Worker) and passive surveillance (by Accredited Social Health Activist, ASHA). About 11.7% of respondents underwent treatment in the local pharmacy or by a local quack. Nearly, 44% of respondents had malaria episodes in previous year i.e., 2017, and about 67.5% got infection in the last 2–3 years (2015-17). The knowledge about malaria is transmitted by mosquito bite was found correct in 78.3% of the respondents, while 32.5% did not know or refused to answer. It was also observed that 72.5% of the respondents knew the preventive measures for malaria, but only 56.7% adopted the preventive measures. The primary reason for not using anti-mosquito measures was ignorance (44.2%), followed by the financial constraints (15.8%) of respondents, or they did not care (17.3%) (Table 3). The anti-mosquito measures generally adopted by the respondent were the usage of bed nets, mosquito coil, mosquito repellents, and burning dried neem leaves.

**Figure 5:**
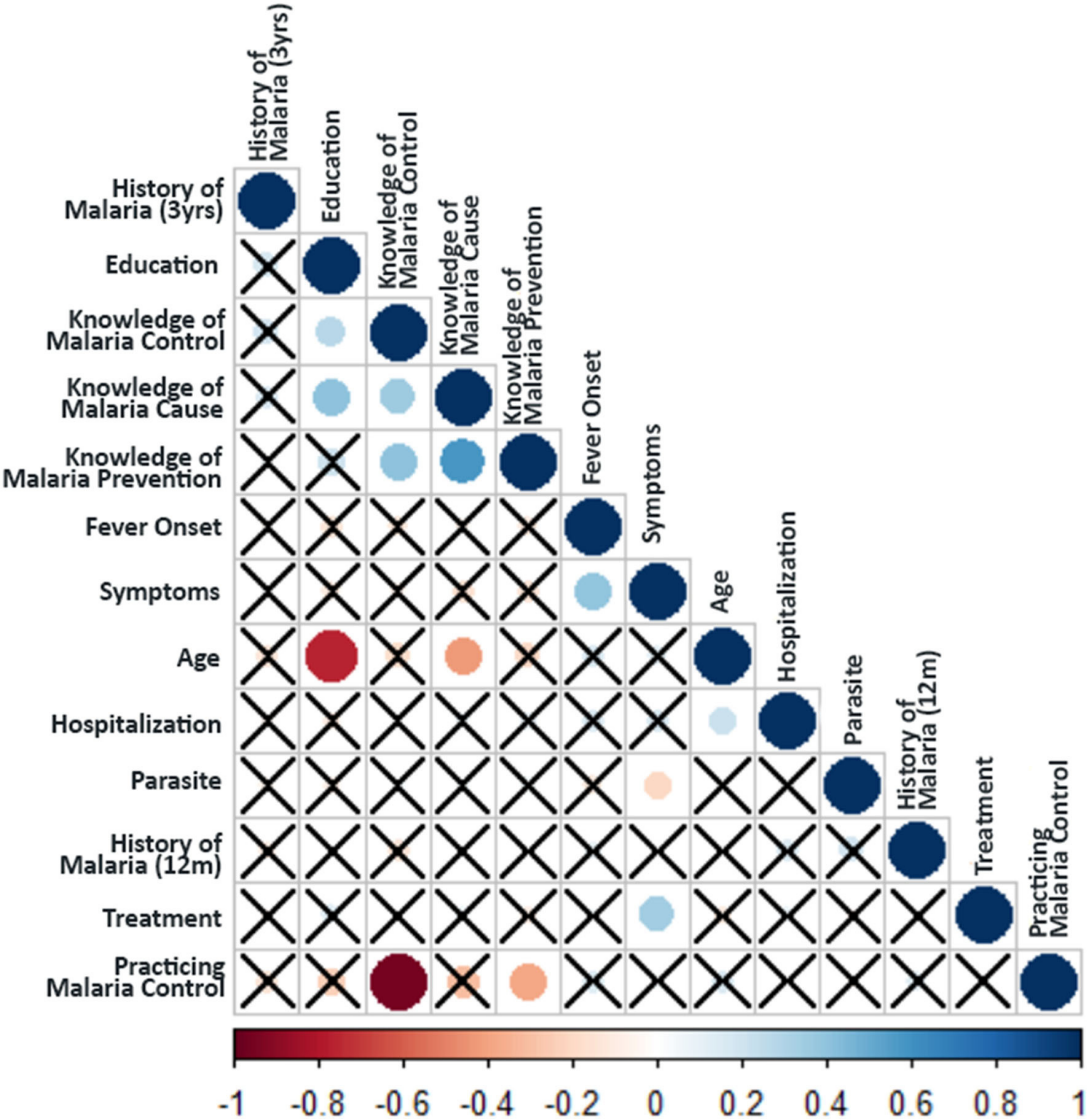
Spearman's rank Correlation test between the KAP parameters. Crosses indicate no relationship between the variables (i.e., non-significant association *p*-value > 0.1). The color of circles in the plot indicates the magnitude of the association (as per the scale bar), whereas the size of the circle indicates the significance (larger size indicates higher significance i.e., lower *p*-value).

We further cross tabulated the variables studied in KAP survey and chi-square test was performed to provide magnitude of the relationship between different parameters in the KAP survey. A correlation matrix was plotted (Figure 5) between the KAP variables, which showed a significant association of age with education (r_s_ = −0.66; *p* < 0.001), event of hospitalization (r_s_ = 0.25; *p* = 0.004; χ^2^ = 9.59; *p* = 0.072) and knowledge about malaria cause (r_s_ = −0.42; *p* < 0.001; χ^2^ = 21.80; *p* < 0.001). The type of malaria symptoms like body pain, fever, chill, headache and vomiting had strong association with the fever onset time (r_s_ = 0.37; *p* < 0.001; χ^2^ = 97.72; p <0.001), parasite species (*P. falciparum*/ *P. vivax*) (r_s_ = −0.25; *p* = 0.007; χ^2^ = 18.41; *p* = 0.003) and their treatment seeking behavior (r_s_ = 0.31; *p* = 0.001). The data showed that knowledge about malaria cause (r_s_ = 0.37; *p* < 0.001; χ^2^ = 20.63; *p* < 0.001) and its prevention (r_s_ = 0.18; *p* = 0.050), as well as malaria control practices (r_s_ = 0.26; *p* = 0.005; χ^2^ = 9.41; *p* = 0.024) were higher in educated respondents. Further, malaria control practices were followed by only those who had knowledge about the cause of malaria (r_s_ = 0.36; *p* < 0.001; χ^2^ = 13.50; *p* < 0.001) and its prevention (r_s_ = 0.40; *p* < 0.001; χ^2^ = 17.71; *p* < 0.001) (Supplementary Table 7 and Figure 5).

**Figure 6:**
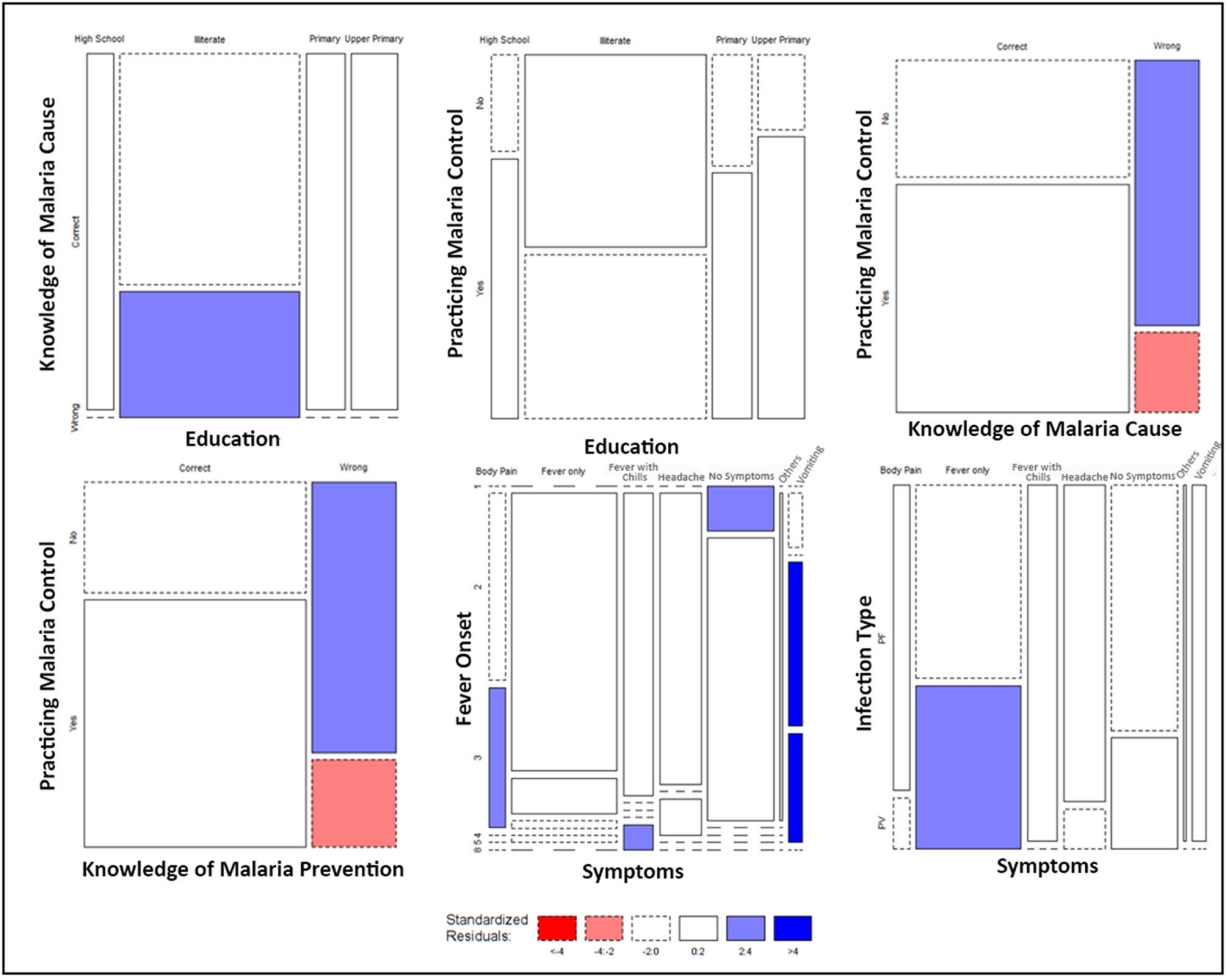
Mosaic Plots of significantly associated KAP variables. Width of the boxes indicates the proportion of respondents, whereas the height of the boxes indicates the proportion of each row variable. Colors indicate the range of the standard residuals estimated by calculating the difference between the observed values and expected values due to random chance.

The significant associations between the responses of KAP data were further mapped on a mosaic plot (Figure 6). Though education appears to significantly influence the knowledge and practices followed by the respondents for malaria control; still, the magnitude of the association between education and practicing malaria control was found to be relatively low (Supplementary Table 7). The practice of malaria control was also significantly associated with the knowledge of malaria cause and its prevention. A large proportion of the respondents that lacked knowledge about malaria cause and its prevention did not practice malaria control, and the estimated residuals were significantly high (Figure 6). The symptoms experienced by the respondents were found to be significantly associated with the number of days that took place for the onset of fever and the type of infection (*P. falciparum*/*P. vivax*). We found that the respondents with a lengthened period for the onset of fever were more likely to experience symptoms such as vomiting and body ache (Figure 6). Furthermore, it was found that patients with *P. vivax* infection were significantly more likely to experience fever only, whereas the majority of the patients that experienced fever with chills had *P. falciparum* infection (Figure 6).

## Discussion

This study comprised investigations related to previous knowledge on malaria epidemiology, entomological survey, meteorology and Knowledge, Attitude and Practices related to malaria control and prevention in the affected region. The study reinforced the important role of Information Education Communication (IEC) and Behavioral Change Communication (BCC) activities in malaria containment. IEC activities include development of communication materials/tools to promote positive behaviors toward disease management like encouraging people to sleep under insecticide-treated bed nets (26), and BCC activities include community mobilization, use of folk/mass media to provide health education (27). The community healthcare workers are trained for malaria control and interpersonal communication through face-to-face education, group teaching or sessions to educate community and influence the behavior of participants toward management and control of malaria (28). The factors affecting the performance of IEC/BCC activities include behavior of the community toward treatment seeking, social behavior like sleeping under bed nets/adherence to the prevention measures, migrant population/forest workers, adequate supply of long-lasting insecticidal nets/IEC material and mode of information dissemination like mass media/personal communication (29). While, education level influenced the adoption of malaria control measures of an individual, the knowledge on the cause of malaria and its prevention, even among lower educated population groups, had much stronger effect on behavioral change. As a result, a stronger attention needs to be paid to sensitize the community by promoting malaria awareness programs for adopting malaria control practices in North-Eastern India.

Malaria remains as one of the most widespread infectious disease within the subtropical and tropical regions in the globe, particularly in Africa (1) and NE India (13). Malaria transmission in NE India presents a major epidemiological challenge, particularly in forest areas and among tribal populations. The populations's susceptibility to malaria infection increases with high vector density, poor socio-economic condition, lack of awareness in the community about malaria, and abundant presence of parasites in the environment (30). About 60% of malaria cases in India are attributed to tribal communities (30). Many tribal groups inhabit NE India with distinct cultural, social, and occupational behavior patterns, like treatment seeking behavior, forest farming, agriculture work, poor socio-economic status, cattle herding, avoiding sleeping under bed nets, low education status, living in bamboo houses and low monthly income, all of which may favor malaria transmission (31). We observed in this study that 74% of the affected population of the community was tea tribes, and about 90% of malaria cases were reported among the tea tribes (data not shown). Therefore, the main information campaigns need to be targeted according to the needs and customs of tea tribe populations. The previous reports from this region also suggested high positivity rate (~42%) among tea tribes suggesting the vulnerability of this population group to malaria (32). The villages at the fringe of Dhunsiri tea garden that comes under the Dolonibasti sub-center have been more affected than the other sub-center areas in 2009 (32). It was also observed that the housing condition of the tea tribes at the fringe of the tea garden is of kutcha type with inadequate drainage facility that leads to the risk of high malaria transmission.

*Anopheles minimus* s.l. was the primary vector with high density (PMHD 1.70) followed by *An. dirus* s.l. and *An. fluviatilis* s.l. in the current study. The resting habits of *An. minimus* s.l. in human dwellings were found to be hanging clothes. Therefore, the Indoor Residual Spray (DDT 50%) has been of limited effect, and, due to poor housing condition, the spraying is also not effective. *An. minimus* s.l. is a highly competent malaria vector responsible for *P. falciparum* malaria outbreaks in the forest fringe and foothill areas of NE India (33). Though the vector may be susceptible to DDT, available literature suggests behavioral resistance and avoidance of both DDT and LLIN in the mosquito vector (34).

This study observed that fever was the most common symptom of malaria among the respondents (38.33%), followed by asymptomatic cases (24.17%). Asymptomatic cases include those individuals that carry the malaria parasites without showing any clinical symptoms, but remain infectious to mosquitoes and continue to spread the disease (35). Therefore, they pose a potential threat to malaria elimination efforts. Among the respondents, 44.2% had malaria episodes in the past year, i.e., 2017, and about 67.5% had malaria episodes in the last 2–3 years (2015-17). This suggested that the population residing in the Dolonibasti sub-center was continuously exposed to malaria episodes, and the region is endemic for sustained malaria transmission (32). More than 70% of respondents knew malaria's cause and preventative measures. However, only 56.7% adopted the preventive measures, and 43.3% of respondents did not use any preventive measures because of many reasons, including poverty (15.77%), ignorance (44.23%) and not caring (17.31%). Previous reports from NE India also emphasized the role of inadequate interventions and treatment seeking behavior of the community (36).

During this study, all the respondents received long-lasting insecticidal nets in 2016. Two rounds of Indoor residual spray (DDT 50%) were done (April to August) by the health department of Assam state to control malaria transmission in the area. However, due to loss or damage of long-lasting insecticidal nets, 6.7% of respondents were not using it as protection. The previous studies have analyzed the nationally representative data obtained from the National Family Health Survey 2015–2016 of India, aand reported that substance use such as alcohol consumption, tobacco smoking and smokeless tobacco are significant in NE India (70.8%) in comparison to elsewhere in India (50.0%). They also reported that out of 10.2 million substance users in India, 6.7 million belong to Assam state (37). An average alcohol consumption among men and women residing in alcohol hot spot districts of Assam state, one of the district is Udalguri district, showed prevalence of 35.6 and 6.9%, respectively (38). Overdrinking of alcohol is a common practice among the population of the Dolonibasti sub-center, and it could be the reason for ignorance or not using any anti-mosquito measures. This study also aimed to assess the impact of meteorological factors on malaria persistence. We found that most malaria cases appeared between April and September and peaked at June-July, corresponding to the monsoon season. This season was characterized by optimum rainfall and suitable temperatures, which promote vector breeding and contribute to the increase in malaria cases, which is evident from the scatter plot between monthly seasonality in meteorological parameters and malaria (Figure 4). This observation was supported by the significant and robust association between monthly meteorological variables (temperature, rainfall and relative humidity), and malaria incidence evaluated in this study. A previous study in NE India reported temperature and rainfall as the most influencing factors for high rate of malaria transmission (39). As most of the cases predicted in the month of June; therefore, an enhanced information campaign could be launched in the beginning of July to reinforce control measures during years, which seems to be favorable for malaria mosquito breeding. Though rainfall broadly supports malaria transmission in NE India, annual cases of malaria and meteorological parameters revealed (Supplementary Table 4) that the years which experienced more than 2000 mm of rainfall had lower malaria incidence (less than 1000 cases in 2012, 2013 and 2017). The study area lies in the foothill region that normally experiences sufficient amount of rainfall to support vector breeding, and is prone to flooding, leading to flushing vector habitats during excess rainfall years. This threshold may be attributed to the likely reason for the non-significant negative association observed between annual rainfall and malaria incidence in the present study. In 2018, when a sharp rise in malaria cases followed, the region experienced an annual rainfall of almost 1,700 mm, sufficient to support vector breeding but insufficient to lead to habitat flushing. Yearly trends in temperature and humidity also did not show any significant association with annual malaria cases, indicating that annual trends in meteorological parameters did not play an essential role in abrupt rise of malaria cases in 2018. Our observations are in accordance to the previous findings from Dispur, Assam state, where no association was observed between annual rainfall determinants and malaria incidence (40).

While analyzing the role of Knowledge, Attitude and Practices related to malaria in the Dolonibasti sub-center, we found a significant correlation between education with the knowledge and practices followed by the community for malaria control, but the magnitude of association was weak. This shows that formal education can help improve the malaria control practices among the community but only to a certain extent, and more concerted efforts need to be made to educate the community regarding malaria control practices. Older people were found to be less educated and therefore had lesser knowledge of malaria cause than the younger ones. The previous studies have assessed the influence of demographic and socio-economic factors, knowledge, awareness and education of community on incidence of malaria. They found that lower income, living in bamboo houses, distance to health sub-center, knowledge and awareness about malaria, number of mosquito bites per day and use of bed nets were positively associated with malaria occurrence (31). The result suggests that increased efforts on Information Education Communication and Behavioral Change Communication campaigns are needed to strengthen the efforts to control malaria in the region (36). The disease burden was much higher among tribes because of their living standards, high population density, poor drainage facility and not using protective measures from mosquito bites. Repeated infections, poor drug compliance and treatment seeking behavior causes non-clearance of parasites and develop drug resistance, contributing to asymptomatic infection in the community (41). Although, regular vector control measures were undertaken in the Dolonibasti sub-center, the community's poor housing conditions and living standards made them more vulnerable to malaria. To prevent future outbreaks, systematic monitoring of environmental risk factors, vector prevalence, and disease surveillance should be carried out at the village level. Involvement of the community in various vector control strategies and source reduction must be carried out. In addition, all symptomatic and asymptomatic carriers should be treated by intensifying the early detection and treatment processes to reduce the risk of transmission. Despite our efforts to summarize the factors associated with malaria persistence in the outbreak region, this study has some limitations, such as the dipping method used for larvae collection may vary from one person to another. Socio-economic and demographic details of participants could be more elaborate, and the exact coordinates of the household were not collected to point out the malaria hotspot. Nevertheless, this was an outbreak investigation with the limited preparedness time for such detailed investigations.

In summary, an overall decline in malaria transmission was observed between 2011-2017 followed by a distinct upsurge in 2018. Malaria persistence continued to play a major role in illness in the Dolonibasti sub-center. High vector density, high parasite positivity in mosquitoes, presence of asymptomatic human carriers compounded by meteorological factors along with topography and geographical location of the region were the main driving factors for malaria persistence. In addition, community knowledge and perception toward malaria likely to have played a significant role in consistent malaria cases in the region. Information Education Communication and Behavioral Change Communication need to be strengthened in areas with persistent malaria.

## Data Availability Statement

The original contributions presented in the study are included in the article/Supplementary Material. This data was a part of investigations performed by the Udalguri district unit of NVBDCP, Government of India in response of malaria outbreak at Dolonibasti health sub-centre. Further inquiries can be directed to the corresponding author/s.

## Ethics Statement

Ethical review and approval was not required for the study on human participants in accordance with the local legislation and institutional requirements. Written informed consent from the participants or their legal guardian/next of kin was not required to participate in this study in accordance with the national legislation and the institutional requirements.

## Author Contributions

RA, AS, and AB wrote the first draft of the manuscript. RA and AK were involved in study conception and design. HS and SH contributed substantially to analysis and interpretation of the data and statistical modeling. RA and HSu executed the field investigations. AK and HSu coordinated the field operations and logistic arrangements. HSh, MT, PP, KS, JKC, and JS drafted the final version of the manuscript, critically reviewed the manuscript. All authors provided intellectual inputs, reviewed and approved this manuscript.

## Conflict of Interest

The authors declare that the research was conducted in the absence of any commercial or financial relationships that could be construed as a potential conflict of interest.

## Publisher's Note

All claims expressed in this article are solely those of the authors and do not necessarily represent those of their affiliated organizations, or those of the publisher, the editors and the reviewers. Any product that may be evaluated in this article, or claim that may be made by its manufacturer, is not guaranteed or endorsed by the publisher.
