# Supplementary material for: Moderate Rainfall and High Humidity During the Monsoon Season, Negligence in Using Malaria Protection Methods and High Proportion of Mild Symptomatic Patients Were the Driving Forces for Upsurge of Malaria Cases in 2018 Among Tea Tribe Populations in Endemic Dolonibasti Health Sub-center, Udalguri District, Assam State, North-East India
Source: Front Med (Lausanne). 2022 Jun 30;9:913848. doi: 10.3389/fmed.2022.913848 (PMC9280886; doi:10.3389/fmed.2022.913848)
Supplement: Supplementary file 1 [file Data_Sheet_1.docx]

**Supplementary File:** Moderate rainfall and high humidity during the monsoon season, negligence in using malaria protection methods and high proportion of mild symptomatic patients were the driving forces for upsurge of malaria cases in 2018 among tea tribe populations in endemic Dolonibasti health sub-centre, Udalguri district, Assam state, North-East India

Supplementary Table 1: Malaria epidemiological data of Udalguri district (2008−2021).

| **Year** | **Population** | **BSC/E** | **Total Positive** | **Pf** | **PV** | **ABER** | **API** | **SPR** | **Pv%** | **Pf%** | **Death** |
| --- | --- | --- | --- | --- | --- | --- | --- | --- | --- | --- | --- |
| **2008** | 929213 | 113039 | 13510 | 3219 | 10291 | 12.17 | 14.54 | 11.95 | 76.17 | 23.83 | 5 |
| **2009** | 872888 | 126660 | 14376 | 4140 | 10236 | 14.51 | 16.47 | 11.35 | 71.20 | 28.80 | 0 |
| **2010** | 867842 | 163446 | 13767 | 3188 | 10579 | 18.83 | 15.86 | 8.42 | 76.84 | 23.16 | 2 |
| **2011** | 873262 | 156108 | 9584 | 3189 | 6395 | 17.88 | 10.97 | 6.14 | 66.73 | 33.27 | 0 |
| **2012** | 878552 | 186644 | 7740 | 2096 | 5644 | 21.24 | 8.81 | 4.15 | 72.92 | 27.08 | 0 |
| **2013** | 895171 | 145632 | 3874 | 1231 | 2643 | 16.27 | 4.33 | 2.66 | 68.22 | 31.78 | 0 |
| **2014** | 896469 | 119491 | 2896 | 1635 | 1261 | 13.33 | 3.23 | 2.42 | 43.54 | 56.46 | 0 |
| **2015** | 901590 | 92735 | 2386 | 1598 | 788 | 10.29 | 2.65 | 2.57 | 33.03 | 66.97 | 0 |
| **2016** | 901511 | 79724 | 1770 | 1139 | 631 | 8.84 | 1.96 | 2.22 | 35.65 | 64.35 | 0 |
| **2017** | 910261 | 85062 | 1129 | 845 | 284 | 9.34 | 1.24 | 1.33 | 25.16 | 74.84 | 0 |
| **2018** | 909442 | 67866 | 2371 | 1907 | 464 | 7.46 | 2.61 | 3.49 | 19.57 | 80.43 | 0 |
| **2019** | 909442 | 103096 | 372 | 261 | 111 | 11.34 | 0.41 | 0.36 | 29.84 | 70.16 | 0 |
| **2020** | 913334 | 113406 | 90 | 51 | 39 | 12.42 | 0.10 | 0.08 | 43.33 | 56.67 | 0 |
| **2021** | 918644 | 75708 | 8 | 3 | 5 | 8.24 | 0.01 | 0.01 | 62.50 | 37.50 | 0 |

BSC/E, Blood Slide Collection/Examination; ABER, Annual Blood Examination Rate; API, Annual Parasitic Incidence; SPR, Slide Positivity Rate; Pv, *Plasmodium vivax*; Pf, *Plasmodium falciparum*

Supplementary Table 2: Malaria epidemiology of Orang Block PHC

| **Year** | **Population** | **BSC /BSE** | **Total Positive** | **Pf** | **PV** | **ABER** | **API** | **SPR** | **Pv%** | **PF%** | **Death** |
| --- | --- | --- | --- | --- | --- | --- | --- | --- | --- | --- | --- |
| **2008** | 234074 | 32857 | 1851 | 1602 | 249 | 14.04 | 7.91 | 5.63 | 13.45 | 86.55 | 3 |
| **2009** | 206836 | 34703 | 2201 | 1708 | 493 | 16.78 | 10.64 | 6.34 | 22.40 | 77.60 | 0 |
| **2010** | 198821 | 45019 | 968 | 842 | 126 | 22.64 | 4.87 | 2.15 | 13.02 | 86.98 | 1 |
| **2011** | 200144 | 39933 | 1608 | 1542 | 66 | 19.95 | 8.03 | 4.03 | 4.10 | 95.90 | 0 |
| **2012** | 208617 | 34532 | 838 | 770 | 68 | 16.55 | 4.02 | 2.43 | 8.11 | 91.89 | 0 |
| **2013** | 232018 | 26815 | 559 | 546 | 13 | 11.56 | 2.41 | 2.08 | 2.33 | 97.67 | 0 |
| **2014** | 232228 | 29982 | 1425 | 1163 | 262 | 12.91 | 6.14 | 4.75 | 18.39 | 81.61 | 0 |
| **2015** | 235752 | 22039 | 1683 | 1240 | 443 | 9.35 | 7.14 | 7.64 | 26.32 | 73.68 | 0 |
| **2016** | 233713 | 19282 | 1458 | 1003 | 455 | 8.25 | 6.24 | 7.56 | 31.21 | 68.79 | 0 |
| **2017** | 235163 | 18822 | 965 | 762 | 203 | 8.00 | 4.10 | 5.13 | 21.04 | 78.96 | 0 |
| **2018** | 238435 | 16881 | 2201 | 1782 | 419 | 7.08 | 9.23 | 13.04 | 19.04 | 80.96 | 0 |
| **2019** | 238435 | 24057 | 314 | 232 | 82 | 10.09 | 1.32 | 1.31 | 26.11 | 73.89 | 0 |
| **2020** | 238724 | 23549 | 73 | 43 | 30 | 9.86 | 0.31 | 0.31 | 41.10 | 58.90 | 0 |
| **2021** | 239123 | 18037 | 8 | 3 | 5 | 7.54 | 0.03 | 0.04 | 62.50 | 37.50 | 0 |

BSC/E, Blood Slide Collection/Examination; ABER, Annual Blood Examination Rate; API, Annual Parasitic Incidence; SPR, Slide Positivity Rate; Pv, *Plasmodium vivax*, Pf, *Plasmodium falciparum*

Supplementary Table 3: Per Man Hour Density of Anophelines and Culicines collected from the villages under Dolonibasti sub-centre

| Mosquitoes species | Number of mosquitoes (n=1005) | Per Man Hour Density |
| --- | --- | --- |
| *Anopheles annularis* s.l. | 12 | 0.30 |
| *Anopheles ahomi* s.l. | 9 | 0.23 |
| *Anopheles barbirostris* s.l. | 50 | 1.25 |
| *Anopheles culicifacies* s.l. | 13 | 0.33 |
| *Anopheles dirus* s.l. | 17 | 0.43 |
| *Anopheles fluviatilis* s.l. | 7 | 0.18 |
| *Anopheles hyrcanus* s.l. | 14 | 0.35 |
| *Anopheles minimus* s.l. | 68 | 1.70 |
| *Anopheles nivipus* s.l. | 15 | 0.38 |
| *Anopheles subpictus* s.l. | 21 | 0.53 |
| *Anopheles splendidus* s.l. | 4 | 0.10 |
| *Culicines (Culex* s.l.*, Mansonia* s.l.*, Aedes* s.l.*, Armigeres* s.l.*)* | 775 | 1.38 |

Supplementary Table 4: Annual trends of malaria incidence and meteorological parameters in the Dolonibasti sub-centre.

| Year | Cases | API | SPR (%) | Temp (°C) | Rf | RH |
| --- | --- | --- | --- | --- | --- | --- |
| 2011 | 1100 | 160.89 | 18.76 | 23.61 | 1633.93 | 75.79 |
| 2012 | 753 | 109.48 | 18.02 | 24.03 | 2276.78 | 75.94 |
| 2013 | 407 | 58.04 | 12.30 | 24.57 | 1920.60 | 75.72 |
| 2014 | 1067 | 147.70 | 14.78 | 24.62 | 1758.10 | 75.01 |
| 2015 | 1326 | 165.87 | 22.43 | 24.56 | 2150.72 | 77.74 |
| 2016 | 1389 | 172.05 | 24.65 | 25.00 | 1785.41 | 78.93 |
| 2017 | 934 | 114.60 | 23.10 | 24.74 | 2007.42 | 75.93 |
| 2018 | 2136 | 260.87 | 32.20 | 24.25 | 1662.01 | 78.48 |

Cases, Malaria cases; API, Annual Parasite Incidence; SPR, Slide Positivity Rate; Temp, Temperature; Rf, Rainfall; RH, Relative Humidity

Supplementary Table 5: Correlation between annual trends in climatic parameters and incidence of malaria in the Dolonibasti sub-centre (2011 – 2018)

|  | **Temperature**  **r_s_** | **Rainfall**  **r_s_** | **Relative Humidity**  **r_s_** |
| --- | --- | --- | --- |
| **Cases** | 0.024  *(0.955)* | -0.476  *(0.233)* | 0.690  *(0.058)* |
| **API** | 0.024  *(0.955)* | -0.476  *(0.233)* | 0.690  *(0.058)* |
| **SPR** | 0.167  *(0.693)* | -0.190  *(0.651)* | 0.833  *(0.010)* |

Cases=Malaria cases, API=Annual Parasite Incidence, SPR=Slide Positivity Rate, r_s_=Spearman’s rank correlation, Parenthesis=p-value

Supplementary Table 6: Monthly seasonality in climatic parameters and incidence of malaria in the Dolonibasti sub-centre (2013 – 2018)

|  | **Temperature**  **r_s_** | **Rainfall**  **r_s_** | **Relative Humidity**  **r_s_** |
| --- | --- | --- | --- |
| **Malaria Cases** | 0.735  *(*<0.001*)* | 0.617  *(*<0.001*)* | 0.631  *(*<0.001*)* |

r_s_=Spearman’s rank correlation, Parenthesis=p-value

Supplementary Table 7: Chi-square test of independence between different KAP parameters

| **Dependent Variable** | **Independent Variable** | **Chi Square value** | **Cramer's V** | **p-value** |
| --- | --- | --- | --- | --- |
| Education | Knowledge of Malaria Cause | 20.633 | 0.415 | <0.001 |
|  | Knowledge of Malaria Prevention | 5.755 | 0.219 | 0.150 |
|  | Practicing Malaria Control | 9.412 | 0.28 | 0.024 |
| Age | Knowledge of Malaria Cause | 21.8 | 0.426 | <0.001 |
|  | History of Malaria (12m) | 3.87 | 0.18 | 0.460 |
|  | History of Malaria (3yrs) | 3.224 | 0.164 | 0.524 |
| Symptoms | Fever Onset | 97.72 | 0.404 | <0.001 |
|  | Infection Type | 18.408 | 0.392 | 0.003 |
|  | History of Malaria (12m) | 9.795 | 0.286 | 0.122 |
|  | History of Malaria (3yrs) | 5.313 | 0.21 | 0.535 |
| Practicing Malaria Control | Knowledge of Malaria Cause | 13.554 | 0.356 | <0.001 |
|  | Knowledge of Malaria Prevention | 17.709 | 0.403 | <0.001 |
| Hospitalization | Age | 9.588 | 0.283 | 0.072 |
|  | Fever onset | 18.928 | 0.397 | 0.096 |
|  | Infection type | 0 | 0.08 | 1.000 |
|  | History of malaria (12m) | 0.784 | 0.146 | 0.193 |
|  | History of malaria (3yrs) | 0.000 | 0.049 | 0.546 |
| Treatment done at | Education | 8.164 | 0.261 | 0.226 |
|  | Knowledge of cause of Malaria | 1.15 | 0.098 | 0.563 |
|  | Knowledge of malaria prevention | 2.025 | 0.13 | 0.363 |
|  | Adopting malaria control | 0.023 | 0.014 | 0.989 |
| Fever Onset | Age | 195.168 | 0.57 | 0.760 |
|  | Infection type | 1.921 | 0.127 | 0.860 |
|  | History of Malaria (12 m) | 9.779 | 0.285 | 0.082 |
|  | History of Malaria (3 yrs) | 7.959 | 0.258 | 0.159 |
